# Interactive Effects of Dietary Starch Level and Ingredient Grinding Size on Growth, Intestinal Health and Liver Condition of Juvenile Giant Grouper (*Epinephelus lanceolatus*)

**DOI:** 10.1155/anu/3101205

**Published:** 2026-05-09

**Authors:** Albino M. Maveto, Caroline Candebat, Adrien F. Marc, Simon Kumar Das, Leo Nankervis

**Affiliations:** ^1^ Centre for Sustainable Tropical Fisheries and Aquaculture, College of Science and Engineering, James Cook University, Townsville, 4811, Queensland, Australia, jcu.edu.au; ^2^ AIMS@JCU, James Cook University, Townsville, 4811, Queensland, Australia, jcu.edu.au

**Keywords:** carbohydrate inclusion, feed formulation, grouper nutrition, hepatic and intestinal health, sustainable growth

## Abstract

Giant grouper (*Epinephelus lanceolatus*) is a highly valued aquaculture species known for its rapid growth. Traditionally reared using ‘trash fish’ as a nutrient source, production of giant grouper has outpaced the nutritional knowledge required for optimal production. Equally important is an appropriate application of processing aids that facilitate feed physical properties while also supporting growth and health. In extruded aquafeed, starch provides energy while enhancing durability and expansion, promoting floating properties important for feed management. Grinding raw materials is a process central to feed manufacture, facilitating feed processing. However, the interaction of particle size after grinding and fish production performance characteristics is understudied. To address this, the present study aimed to evaluate the effects of varying dietary wheat starch levels and ingredient grinding on growth, intestinal health and liver condition in juvenile giant grouper. Seven isonitrogenous (~55% crude protein) and isocaloric (~19 MJ/kg) diets were formulated, containing low (9%), medium (12%) and high (15%) wheat starch, with ingredients ground to either 1 or 0.5 mm with an additional very high‐starch diet (15.9%) formulated exclusively at the 0.5 mm grinding size and not tested at 1 mm. After a 6‐week trial, elevation of dietary starch levels above the minimum tested led to increasing inflammation in the posterior intestine, evidenced by a thicker lamina propria and a higher prevalence of eosinophilic granular cells characteristic of intestinal inflammation. Fish fed diets made from ingredients milled to 0.5 mm exhibited lower growth rates and enlarged livers due to elevated glycogen deposition in hepatocytes. This study does not support the practice of elevating starch levels above 9% of the feed, nor does it support grinding of raw materials to 0.5 mm. These results provide valuable insights for formulating nutritionally balanced feeds using wheat starches, supporting the sustainable development of giant grouper aquaculture.

## 1. Introduction

Giant grouper (GG, *Epinephelus lanceolatus*), is an economically important aquaculture species in Southeast Asia which is also emerging in Australia due to its high market value and rapid growth rate under farm conditions [1–5]. Traditionally grown in Southeast Asia with low‐value (‘trash’) fish as a feed input, GG farming has largely developed without formulated feeds [6, 7]. Feeding fish to other fish is limited in sustainable scale, while also posing issues with shelf‐life, inconsistent nutritional profile and increased environmental and biosecurity risks [6, 8, 9].

Where formulated feeds have been implemented, the GG aquaculture industry has largely adopted generic commercial aquafeeds to avoid the issues linked to trash fish. Nevertheless, such feeds may not adequately meet the species‐specific nutritional needs of GG [10]. Thus, defining the nutritional requirements of GG and formulating tailored aquafeeds are crucial for optimising productivity and ensuring sustainable production [11, 12].

Starch is among the key components of extruded aquafeeds, contributing to physical pellet properties, such as water stability [13] and buoyancy [14] in addition to providing an inexpensive energy source that can reduce the inclusion of more costly proteins and lipids [15]. Determining optimal starch inclusion levels is therefore essential for designing diets that satisfy both the technical and nutritional demands of GG. In fish, starch utilisation is influenced by several factors, including source, inclusion level, extrusion processing (e.g. heat, moisture) and ingredient grinding size [16, 17]. For example, gelatinised starch improves digestibility in rainbow trout (*Oncorhynchus mykiss*) [18], while finer grinding increases surface area, enhancing enzymatic access and nutrient availability in vitro [19]. However, most previous studies have evaluated starch inclusion level or processing characteristics independently, rather than considering how these factors may interact within a single dietary framework.

Excess dietary starch has also been linked to proinflammatory and immunological responses in fish [20], particularly in carnivorous species with limited capacity to digest and utilise carbohydrates effectively [21, 22]. In the digestive tract, starch is hydrolysed into glucose, which is absorbed by intestinal enterocytes [23, 24], transported to the liver and used for energy or stored as glycogen in hepatocytes [25–27]. In salmonids, high dietary starch levels result in hepatic glycogen accumulation and liver enlargement [23, 28], which are considered indicative of metabolic stress and compromised welfare. Quantitative histological approaches may elucidate mechanisms of starch influence over fish growth and welfare critical for defining appropriate inclusion levels, similar to other nutrient inputs [29].

Therefore, this study assesses the individual and interactive effects of dietary wheat starch levels and ingredient grinding size in extruded aquafeeds on growth performance, posterior intestinal morphology and liver condition in juvenile GG. By integrating nutritional level and processing‐related particle size within a single experimental design, this study aims to address a clear knowledge gap and provide species‐specific evidence to support more precise feed formulation strategies for GG. This information will inform appropriate levels of wheat starch to guide physical/nutritional qualities of feed without negatively affecting the performance or welfare of GG.

## 2. Material and Methods

### 2.1. Experimental Design and Diet Manufacture

A factorial dose–response experiment was designed to evaluate dietary starch tolerance and the biological effect of feed ingredient grinding before feed manufacture for juvenile GG. Diets with three starch inclusion levels (nominally 9%, 12% and 15%) were formulated (Table 1), and ingredients were either ground to 500 or 1000 µm before extrusion. An additional 15.9% starch diet was produced using only the 0.5 mm grind size to assess the upper threshold of starch tolerance in GG.

**Table 1:** Diet formulation (g kg^−1^, unless otherwise stated) and proximate compostion (% dry matter) of the experimental diets.

Grinding size	1 mm			0.5 mm			
Dietary wheat starch level	Low (9%)	Medium (12%)	High (15%)	Low (9%)	Medium (12%)	High (15%)	Very high (15.9%)
Raw materials (g kg^−1^)							
Rapeseed (35% CP)	20.0	20.0	20.0	20.0	20.0	20.0	20.0
Corn gluten meal (60% CP)	50.1	50.1	50.1	50.1	50.1	50.1	50.0
Fish meal (65%)	400.3	400.3	400.3	400.3	400.3	400.3	400.0
Soy protein concentrate (63% CP)	100.1	100.1	100.1	100.1	100.1	100.1	173.0
Lupin seed meal (39.5% CP)	50.1	50.1	50.1	50.1	50.1	50.1	50.0
Wheat gluten meal (78% CP)	20.0	20.0	20.0	20.0	20.0	20.0	20.0
Soybean meal (45% CP)	100.1	100.1	100.1	100.1	100.1	100.1	0.0
Blood cell meal (spray‐dried)	30.0	30.0	30.0	30.0	30.0	30.0	30.0
Wheat starch	0.0	77.6	155.2	0.0	77.6	155.2	183.0
Fish oil (anchovy)	60.1	60.1	60.1	60.1	60.1	60.1	60.0
Whole wheat	155.2	77.6	0.0	155.2	77.6	0.0	0.0
Grouper vitamin premix^a^	1.0	1.0	1.0	1.0	1.0	1.0	1.0
L‐lysine	3.5	3.5	3.5	3.5	3.5	3.5	3.5
DL‐methionine	7.0	7.0	7.0	7.0	7.0	7.0	7.0
Choline chloride (70% choline)	0.3	0.3	0.3	0.3	0.3	0.3	0.3
DPI ARI fish mineral premix^b^	1.0	1.0	1.0	1.0	1.0	1.0	1.0
Vitamin E‐50	0.2	0.2	0.2	0.2	0.2	0.2	0.2
Yttrium oxide	1.0	1.0	1.0	1.0	1.0	1.0	1.0
Proximate composition (% dry matter unless otherwise stated)							
Starch (calculated^c^)	10.40	13.00	15.51	10.40	13.00	15.51	18.30
Starch (analysed)	9.10	12.60	15.10	8.10	11.90	14.90	15.50
Protein	54.41	55.96	55.61	56.32	56.40	55.22	55.63
Lipid	7.92	7.92	8.09	7.54	7.02	7.36	7.42
Ash	9.30	9.00	9.00	8.90	9.30	9.30	9.00
Moisture (%w/w)	5.70	6.40	4.90	8.60	7.80	4.60	5.20
Gross energy (MJ kg^−1^)	18.90	19.10	19.00	18.90	18.80	19.10	19.10

^a^Grouper vitamin premix (mg/kg of premix): Biotin (B7), 1000; Folic acid (B9), 5000; Niacin (B3), 45000; Pantothenic Acid (B5), 10000; Pyridoxine (B6), 10000; Riboflavin (B2), 20000; Thiamine (B1), 10000; Vitamin B12, 50; Vitamin C, 150000; Vitamin A, 900000; Vitamin D, 0.6; Vitamin K, 10000; Inositol, 250000; Antioxidant, 15000.

^b^Dry matter, 98%; moisture 2%; Ash, 70%; Magnesium, 5.94%; Copper, 1000 mg/kg; Iron, 8000 mg/kg; Manganese, 5000 mg/kg; Selenium, 20 mg/kg; Zinc, 20,000 mg/kg; Iodine, 800 mg/kg; Cobalt, 100 mg/kg.

^c^Dietary starch levels were calculated based on the analysed starch content of whole wheat (67.2%; Symbio Labs, glucoamylase digestion method) and inclusion levels of purified wheat starch.

All diets were isonitrogenous (~55% crude protein) and isocaloric (~19 MJ/Kg gross energy), in accordance with reported optimal protein and protein‐to‐energy requirements for groupers [10, 30]. Other ingredients were adjusted to maintain balanced nutrient profiles. This was achieved by replacing whole wheat with wheat starch in the core six diets. Although whole wheat contains ~10% crude protein, its contribution to total dietary protein is small, and proximate composition analysis shows very similar crude protein contents among diets; therefore, the diets are reported as isonitrogenous. The additional higher‐starch diet was achieved by replacing soybean meal with soya protein concentrate and wheat starch. In this way, we were able to formulate feeds that differed as little as possible, apart from starch which replaced nonstarch carbohydrates. Due to known analytical variability in starch determination of complete feeds, dietary starch levels were calculated based on analysed starch content of whole wheat and inclusion levels of purified wheat starch (Table 1). Specifically, calculated starch values were derived from the analysed starch content of whole wheat (67.2%; Symbio Labs, glucoamylase digestion method) together with the inclusion level of purified wheat starch in each formulation. Starch content of the finished diets was subsequently determined using the same enzymatic method. Differences between calculated and analysed values likely reflect normal variation associated with raw material composition, processing effects during extrusion and inherent analytical variability.

All experimental diets were prepared at James Cook University, Townsville, Queensland, Australia. Prior to pellet extrusion, raw materials were combined according to the diet formulation and mixed in a planetary mixer (Hobart A120, Troy, Ohio) for 15 min. The dry mix was then ground using an SR300 Rotor Beater Mill (Retsch, Haan, Germany) with either a 1000 or 500 µm screen at the mill outlet.

Fresh water was added to make a total moisture content of 28% before being extruded using a 35 mm single‐screw extruder (Telford Smith Engineering, Dandenong, VIC, Australia). Barrel temperatures were set at 100°C, 110°C and 120°C in the three respective zones, and the final melt temperature reached ~120°C before being formed through a 3 mm die. The extrudate was cut to ~4 mm lengths and dried at 60°C to a moisture content of 6%–10% (TD 700‐F Premium Dryer, Thermoline, Wetherill Park, NSW, Australia). Oil was added post‐extrusion to evenly coat the extruded pellets, using a cement mixer, prior to storage at −18°C.

Moisture, crude protein, lipid and gross energy contents were determined by laboratory analyses following AOAC protocols [31–33]. Although starch gelatinisation was optimised during extrusion, it was not directly quantified in this study.

### 2.2. Fish Husbandry, Experimental System and Sampling Procedure

Juvenile GG (40.0 ± 0.95 g) were acclimated in a 1000 L fibreglass tank and fed a commercial aquafeed (Skretting, 3 mm, Nova FF, 40% CP) to satiation for 1 week before the experiment.

The feeding trial was conducted in 21 tanks (500 L fibreglass each) with recirculated seawater (31 ppt, 28°C, technical oxygen and continuous aeration). Fish (*n* = 15 per tank) were weighed (*g* ± 0.01 g), measured (cm ± 0.01 cm) and distributed into tanks in triplicate per diet. Fish were fed to satiation once daily (9:00–10:00 AM) over 42 days. Feed intake (FI) was recorded daily. At the end of the trial, fish were weighed again.

Post‐trial, fish were fasted for 24 h, then anaesthetised with 20 mg/L iso‐eugenol (AQUI‐S, New Zealand). Final weights (FWs) and lengths were measured, and five fish from each tank were dissected for sampling of liver and intestine. Tissues were weighed, and samples of liver and complete intestines were fixed in 10% buffered formalin for histology.

### 2.3. Calculations

Growth and feed utilisation of juvenile GG were assessed as follows [34, 35]:

Condition factor K= Whole body wet weight gTotal length3 cm× 100,

 Weight gain WG% =Final weight g−Initial weight gInitial weight g× 100

Feed intake FI g/fish=Total dry feed distributedNumber of fish 

Specific growth rate SGR %= ln final weight g−ln initial weight gExperimental period days×100

where ln=natural  log.

Feed conversion ratio FCR = Weight of food eaten gWeight gain of fish g ×100

Hepatosomatic index (HSI) [36] and Viscerosomatic index (VSI) [37] were determined according to the following formulae:

Hepatosomatic Index HSI%=Liver weight gWhole fish weight g× 100

Viscerosomatic Index VSI%=Visceral weight g Whole fish weight g× 100

### 2.4. Histological Processing and Staining Process

#### 2.4.1. Tissue Processing/Dehydration

Following fixation, liver and posterior intestine tissues were cut into ~4 mm pieces and placed into cassettes (small, perforated baskets). Tissues in cassettes were then dehydrated using an enclosed tissue processor (Leica HistoCare Pearl. Nussloch, Germany), which increased the alcohol contents consistently from 70% ethanol (one change ‐ 15 min) to 90% ethanol (one change ‐ 15 min) and 100% ethanol (four changes) for a total of 6 h. This process was followed by a tissue clearing phase, consisting of three changes of 100% xylene for a total of 85 min, to displace ethanol in the tissues and remove a substantial amount of fat that would otherwise interfere with paraffin infiltration.

#### 2.4.2. Paraffin Infiltration and Embedding

The cleared tissues were infiltrated with liquid histological paraffin wax at 60°C using an embedding machine (Leica HistoCare Arcadia H, Nussloch, Germany). Further, the tissues were placed in a metallic mould (40 × 24 × 20 mm) and filled with molten wax before being placed on a cold plate to solidify and form a block, using the machine’s cold section (Leica HistoCare Arcadia C, Germany). Finally, each block was cut into 5 µm slices using a manual sectioning microtome (Rotary 3003 PFM, Köln, Germany) and mounted prior to staining.

#### 2.4.3. Tissue Staining

Haematoxylin and eosin (H&E) staining was performed according to Fischer and Jacobson [38] for the histological analysis of tissue and cell structures. Periodic Acid Schiff reaction (PAS) and PAS reaction with diastase digestion (PAS‐D) were performed according to a modified version of Luna [39] for analysis of glycogen deposition. In brief, duplicate sections from each liver sample were taken. One glass slide was digested with a 0.45% diastase solution (Chem‐Supply™, Diastase LR (a‐Amylase), DL026‐100 g) for 1 h, before passing through the PAS procedure.

### 2.5. Quantitative Analysis of Histological Work

Following staining, the slides were examined under light microscopy (Olympus U‐DO3, Japan) and scanned using a digital scanner (Leica, Aperio LV1, Nussloch, Germany) at 10× resolution with three layers and a pixel size of 2.0 µm to generate histological images for posterior analysis.

#### 2.5.1. Intestine Morphometrics

**Figure 1:**
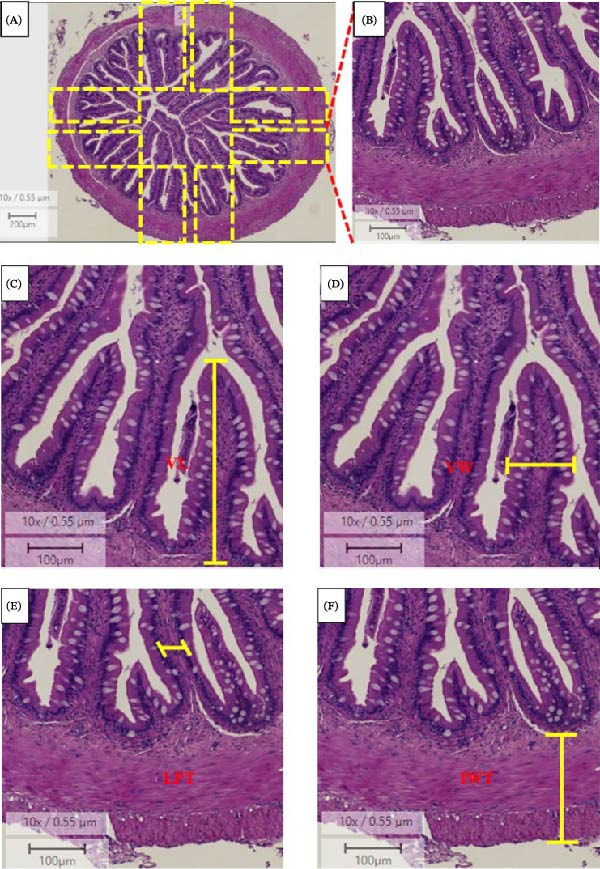
Intestine morphometric measurements. (A)–(B) Systematic uniform random sampling of the field of view (FOV), (C) VL, villus length, (D) VW, villus width, (E) LPT, lamina propria thickness and (F) IWT, intestinal wall thickness.

For each sample, a full image of the posterior intestine was selected using image processing software (Aperio ImageScope v12.4.3.5008, Wetzlar, Germany) (Figure 1). Eight different sites were selected to measure the villus length (Figure 1C), the villus width (Figure 1D), the lamina propria thickness (Figure 1E) and intestinal wall thickness (Figure 1F). The villus length was measured from their base through to their distal tip. Only intact villi were considered. The intestinal wall thickness was measured considering the distance between the tunica serosa and the base of the villus. Eight measurements were taken for each parameter.

#### 2.5.2. Liver Morphometrics

**Figure 2:**
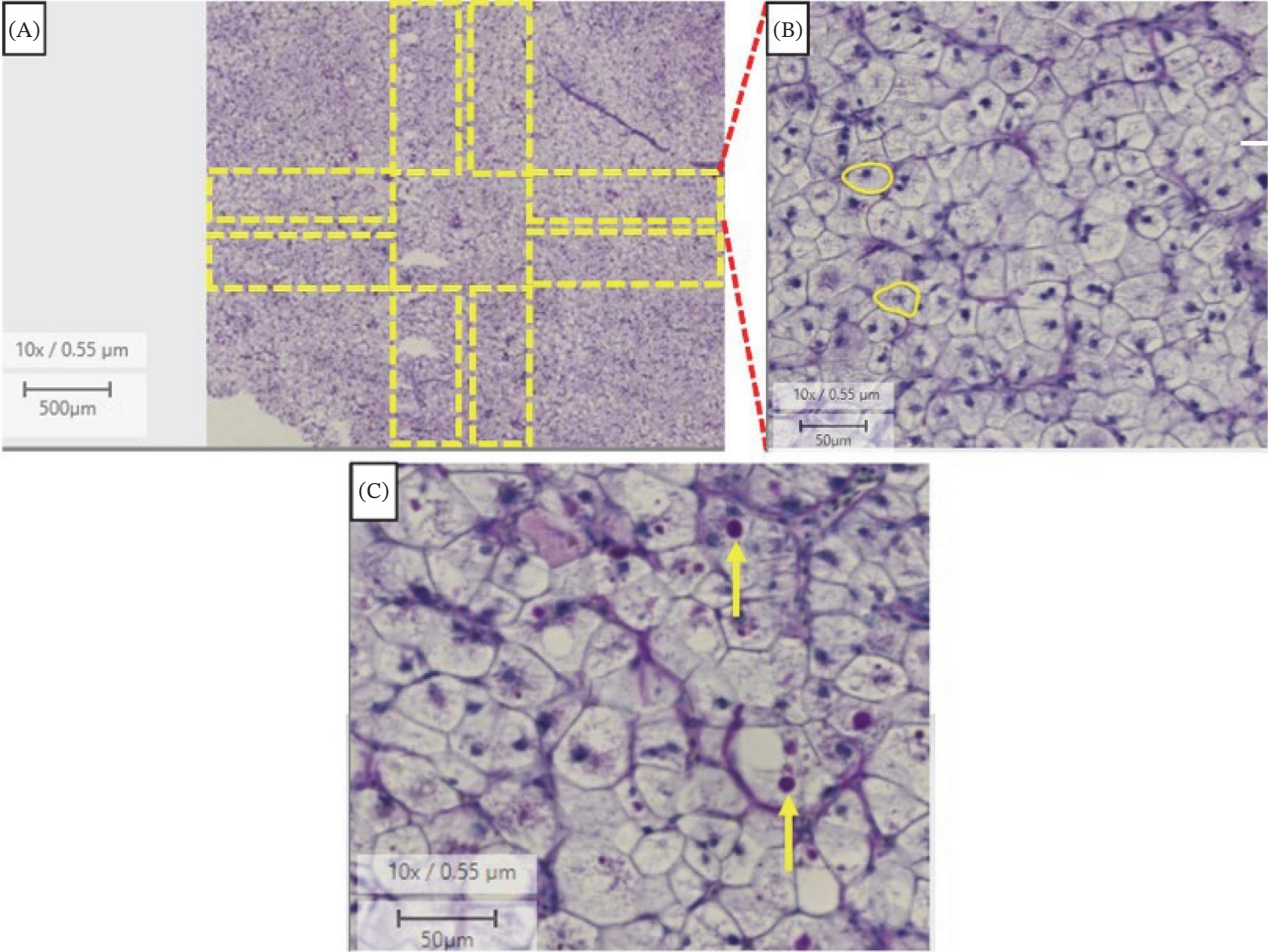
Liver morphometric measurements. (A) Systematic uniform random sampling of field of view (FOV), (B) hepatocyte area and (C) glycogen level.

Glycogen levels in the liver were assessed using non‐digested PAS‐stained sections. The estimation was based on colour metrics obtained from two colour systems: the mean M value from the CYMK colour system and the L value from the Lab colour system (Figure 2). Adobe Photoshop (Adobe Systems, San Jose, CA, USA) was employed to extract these colour values, which served as a relative indicator of glycogen deposition in hepatocytes. Accordingly, glycogen values are expressed in arbitrary units (a.u.) rather than as absolute concentrations.

To quantify hepatocyte area, one field of view (FOV) was randomly selected per sample at a resolution of 500 μm. Within this FOV, eight distinct sections were further chosen to measure the average hepatocyte area at a 50 μm resolution. Image analysis was performed using Aperio ImageScope software (v12.4.3.5008, Wetzlar, Germany).

#### 2.5.3. Statistical Analysis

All data were presented as means ± standard error (SE). Data were analysed for normality (Shapiro–Wilk test) and homogeneity of variance (Levene’s test), and if assumptions were not met, data were log‐transformed. As the number of starch levels differed between grinding sizes, the data were analysed using a general linear model (GLM) in R. Starch level and grinding size were included as fixed factors, together with their interaction. The significance of main effects and interactions was assessed within this framework at *p* < 0.05. When significant differences in starch levels were detected, Tukey’s HSD multiple comparisons were performed within each grinding‐size group to control the family‐wise error rate. Pearson’s correlation analysis was used to determine the relationship between HSI and the hepatocyte area and between villus length and lamina propria thickness. Simple linear regression analyses were conducted to assess the relationships between dietary starch level and hepatocyte area and HSI. Regression models were fitted separately for each grinding size. Model assumptions were evaluated using residual plots, and significant regressions were defined when *p* < 0.05. Coefficients of determination (*R* ^2^) were calculated to assess goodness of fit. R statistical software version 4.1.2 (2021) was used for all statistical analysis.

## 3. Results

### 3.1. Growth and Feed Performance

Growth performance in juvenile GG was influenced significantly by the grinding size of the ingredients (Table 2). Fish fed diets made from coarsely ground (1 mm) ingredients consistently demonstrated higher FW, weight gain (WG) and specific growth rate (SGR) than those fed the finer grinding (0.5 mm) diets. For instance, at the very high‐starch level (15.9%), FW in the 1 mm group reached 182.13 ± 3.7 g compared to 167.82 ± 4.0 g in the 0.5 mm group. This corresponds to a relative WG of 364 ± 9.8% vs. 327.88 ± 11.3%, respectively.

**Table 2:** Growth performance and feed utilisation of juvenile giant grouper fed seven experimental diets containing low, medium, high and very high levels of starch, with two different grinding sizes (1 and 0.5 mm) for 42 days.

Response variables	1 mm			0.5 mm				ANOVA (*p*‐Value)		
	Low	Medium	High	Low	Medium	High	Very‐high	Starch	Size	Size × starch
IW (g)	40.8 ± 1.20	40.74 ± 0.92	39.44 ± 1.02	39.35 ± 1.1	40.31 ± 0.6	39.36 ± 0.8	40.02 ± 0.8	ns	ns	ns
FW (g)	186.64 ± 4.9	172.86 ± 4.5	182.13 ± 3.7	172.87 ± 5.6	172.00 ± 4.4	167.82 ± 4.0	164.05 ± 5.1	ns	^∗^	ns
K	2.26 ± 0.1	2.25 ± 0.0	2.39 ± 0.1	2.22 ± 0.0	2.32 ± 0.1	2.24 ± 0.0	2.33 ± 0.0	ns	ns	^∗^
WG (%)	360.47 ± 14.1	325.95 ± 11.6	364 ± 9.8	344.63 ± 19.1	329.35 ± 15.2	327.88 ± 11.3	311.91 ± 14.2	ns	^∗^	ns
FI	134.11 ± 1.3^c^	124.57 ± 1.3^b^	124.3 ± 0.6^b^	127.94 ± 1.5^b^	127.52 ± 1.0^b^	117.03 ± 1.6^a^	111.71 ± 0.7^a^	^∗∗^	^∗∗^	^∗∗^
SGR	3.62 ± 0.07	3.44 ± 0.06	3.65 ± 0.04	3.52 ± 0.10	3.45 ± 0.08	3.45 ± 0.06	3.35 ± 0.08	ns	^∗^	ns
FCR	0.93 ± 0.03	0.95 ± 0.02	0.88 ± 0.02	0.98 ± 0.04	0.99 ± 0.04	0.92 ± 0.03	0.93 ± 0.04	ns	ns	ns
HSI	1.68 ± 0.15^a^	2.27 ± 0.17^abc^	2.94 ± 0.23^bc^	2.12 ± 0.12^a^	2.34 ± 0.18^abc^	3.12 ± 0.45^c^	3.22 ± 0.45^c^	^∗∗^	^∗^	ns
VSI	10.20 ± 0.41	10.29 + 0.17	11.4 ± 0.21	10.68 ± 0.18	10.69 ± 0.21	10.83 ± 0.21	10.23 ± 0.75	ns	ns	ns

*Note:* Values are shown as the means ± SE of three replicates groups. Within each grinding size, significant differences among starch levels are denoted with different superscripts (*a–c*), as analysed by two‐way ANOVA followed by Tukey’s post hoc multiple comparison test. Means sharing at least one letter are not significantly different *(* *p*  < 0.05). ns = denotes no significant differences, as analysed by two‐way ANOVA.

Abbreviations: FCR, Feed conversion ratio; FI, Feed intake; FW, Final weight; HSI, Hepatosomatic index; IW, Initial weight; K, Condition factor; SGR, Specific growth Rate; VSI, Viscerosomatic index; WG, Weight gain.

^∗^Denotes significant differences at (*p* < 0.05).

^∗∗^Denotes significant differences at (*p* < 0.001).

SGR was significantly affected by ingredient grinding size (*F* (*1*, *98*) *= 4.65*, p ≤ 0.05), with fish receiving the 0.5 mm diet exhibiting an overall reduction in growth. The lowest SGR (3.35 ± 0.08) was recorded in fish fed the very high‐starch diet (15.9%) with a 0.5 mm grinding size, compared to the highest SGR (3.65 ± 0.04) observed in fish fed the 15% starch diet with a 1 mm grinding size. Although the dietary starch level did not significantly impact SGR (*F* (*3*, *98*) = 1.67,*p*  > 0.05), a downward trend was observed with increasing starch inclusion, especially in the 0.5 mm diet group.

FI was highly responsive to both grinding size and starch level. A significant interaction effect (*F* (*3*, *98*) *= 47.31*, *p*  < 0.001) was noted, with FI progressively decreasing as starch level increased. At a 0.5 mm grind size, FI dropped by 12.7% from 127.94 ± 1.5 g (9% starch diet) to 111.71 ± 0.7 g (very high‐starch diet, 15.9%). Likewise, across all starch levels, fish fed the 0.5 mm grinding‐size diets consistently showed lower FI than those on the 1 mm grinding‐size diets (*F* (*1*, *98*) *= 47.37*, *p*  < 0.001).

Of note, FCR remained statistically unaffected by either grinding size (*F* (*1*, *98*) *= 1.50*, *p*  > 0.05) or starch level (*F* (*3*, *98*) *= 1.81*, *p*  > 0.05). Although minor variations existed (e.g. FCR of 0.88 ± 0.02 in the very high‐starch diet (15.9%), 1 mm grinding‐size group vs. 0.99 ± 0.04 in the medium starch diet (12%), 0.5 mm group grinding size), these differences were not statistically significant.

The condition factor (*K*) ranged between 2.22 and 2.39 across treatments and was not significantly impacted by starch level or grinding size, although a minor interaction effect (*F* (*1*, *98*) *= 4.65*, *p*  < 0.05) was observed.

### 3.2. Intestinal Morphometrics

**Figure 3:**
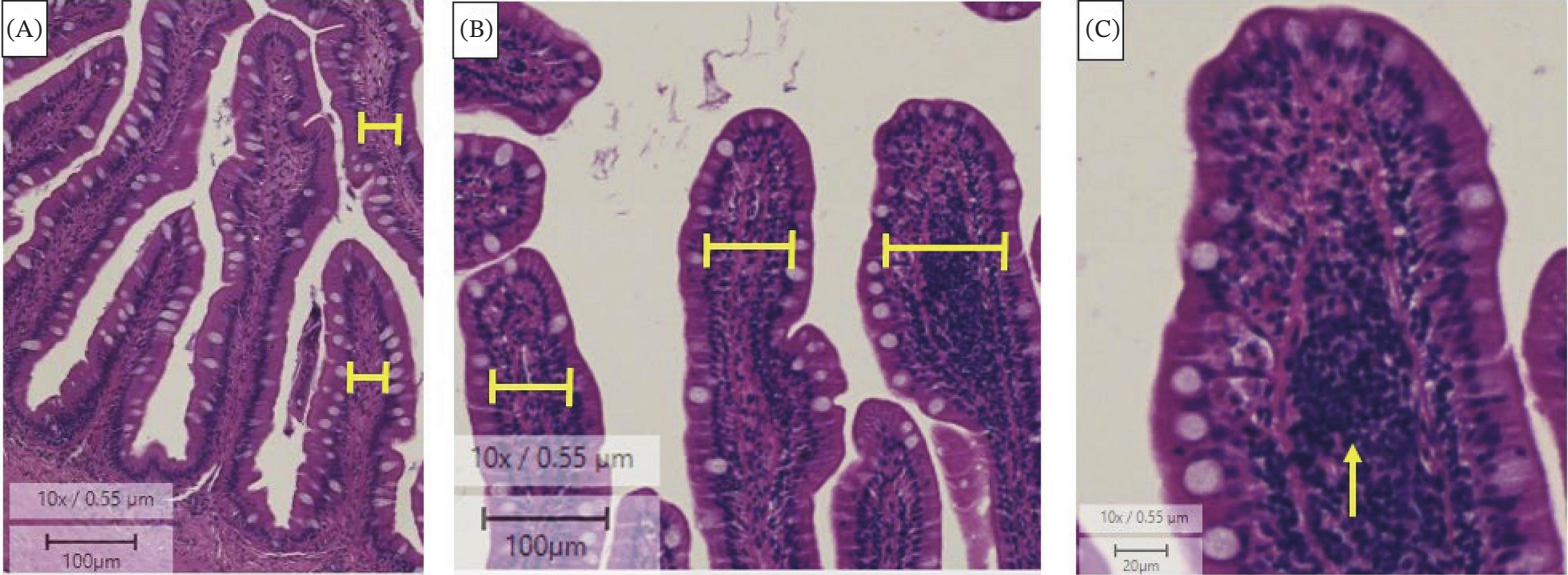
Photomicrograph of the posterior intestine of giant grouper *E. lanceolatus*. (A) the intestine of fish fed a low‐starch diet. (B) Inflammation of the posterior intestine of fish fed very high‐starch diets, showing wider villi and inflamed lamina propria. (C) Infiltration of granular/inflammatory cells into the lamina propria of juvenile giant grouper. Arrow – granular/inflammatory cells.

Dietary starch level, but not ingredient grinding size, had a significant effect on lamina propria thickness (Table 3). An increase in dietary starch level led to a significantly wider lamina propria (F (3, 98) = 6.18, *p*  < 0.001), with the widest lamina propria observed in fish fed the very high‐starch diet (15.9%) (18.37 ± 0.5 µm) compared to those fed the 9% starch diet (13.70 ± 0.4 µm; Table 3, Figure 3A, B). The widening of the lamina propria was visibly associated with an increased prevalence of granular cells (Figure 3C), characteristic of intestinal inflammation. No significant difference was observed in villi length across varying starch levels and grinding sizes (Table 3).

**Table 3:** Intestine and liver morphometrics parameters of juvenile giant grouper fed eight experimental diets containing low, medium, high and very high levels of starch, with two different grinding sizes (1 and 0.5 mm) for 42 days.

Response variable	1 mm			0.5 mm				ANOVA (*p*‐Value)		
	Low	Medium	High	Low	Medium	High	Very‐ high	Starch	Size	Size × starch
VL (μm)	781.8 ± 45.1	831.0 ± 42.5	829.2 ± 38.6	833.6 ± 33.8	837.9 ± 33.7	842.7 ± 42.4	850.3 ± 35.9	ns	ns	ns
VW (μm)	134.6 ± 6.0	128.5 ± 6.2	129.1 ± 9.4	129.9 ± 7.4	139.53 ± 4.8	132.5 ± 7.8	145.2 ± 7.3	ns	ns	ns
LPT (μm)	14.5 ± 0.9^a^	15.2 ± 0.7^ab^	16.0 ± 0.8^ab^	13.7 ± 0.4^a^	16.1 ± 1.1^ab^	17.1 ± 0.8^ab^	18.3 ± 0.50^b^	^∗^	ns	ns
IWT (μm)	412.3 ± 30.9	438.4 ± 41.2	367.2 ± 29.1	436.7 ± 29.5	341.6 ± 29.0	426.6 ± 40.2	376.0 ± 23.7	ns	ns	^∗^
HA (μm^2^)	1002.6 ± 135.9^a^	1147.6 ± 30.6^a^	1324.7 ± 121.1^ab^	894.2 ± 50.6^a^	1302.8 ± 25.3^ab^	1695.3 ± 87.9^bc^	1938.2 ± 207.9^c^	^∗^	^∗^	ns
GL (a.u.)	165.5 ± 6.6^abc^	162.0 ± 4.7^abc^	170.8 ± 5.1^abc^	158.3 ± 3.0^bc^	151.8 ± 4.6^a^	176.3 ± 5.0^bc^	179.8 ± 4.9^c^	^∗^	ns	ns

*Note:* Values are shown as the means ± SE of three replicates groups. Within each grinding size, significant differences among starch levels are denoted with different superscripts (*a–c*), as analysed by two‐way ANOVA followed by Tukey’s post hoc multiple comparison test. Means sharing at least one letter are not significantly different (*p* < 0.05). ns = denotes no significant differences, as analyzed by two‐way ANOVA. *d* = expected wheat starch levels in the diet.

Abbreviations: GL, Relative hepatic glycogen levels estimated from PAS staining intensity (arbitrary units, a.u.); HA, Hepatocyte area; IWT, Intestinal wall thickness; LPT, Lamina propria thickness; VL, Villi length; VW, Villi width.

^∗^Denotes significant differences at (*p* < 0.05).

^∗∗^Denotes significant differences at (*p* < 0.001).

### 3.3. Liver Morphometrics

**Figure 4:**
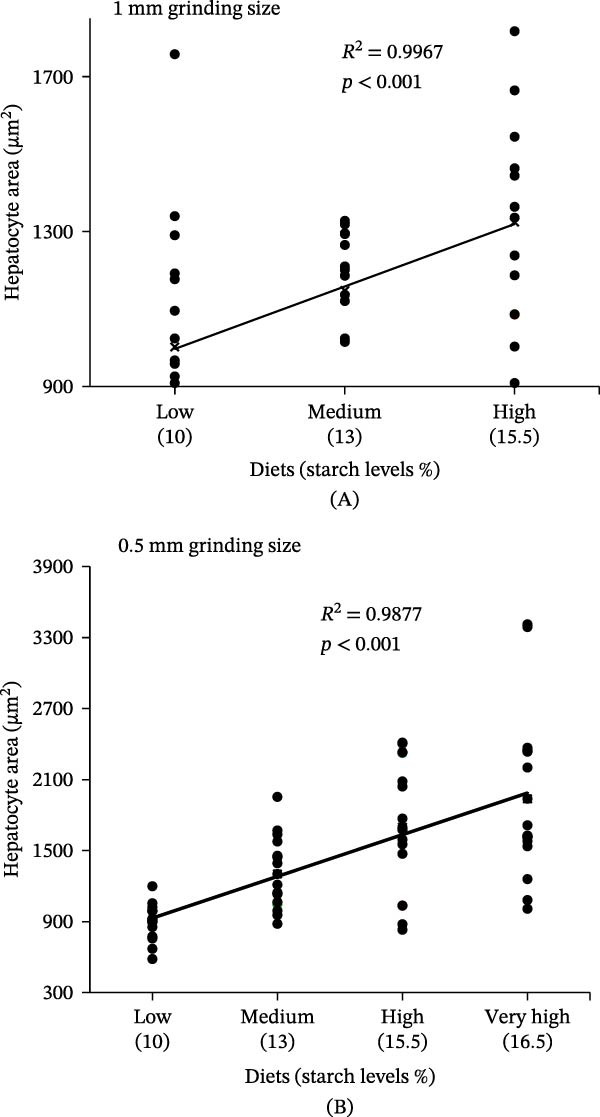
Correlation between hepatocyte area and dietary starch levels in juvenile giant grouper fed diets produced using (A) 1 mm and (B) 0.5 mm ingredient grinding sizes.

Morphometrics analysis of the liver showed a significant increase in the hepatocyte area with higher dietary starch levels (*F* (3, 98) = 17.92, *p*  < 0.001; Table 3, Figure 4). The largest hepatocyte area (1938.18 ± 207.97 µm^2^) was observed in fish fed the very high‐starch diet (15.9%), which was significantly larger than that in fish fed the 9% starch diet (894.15 ± 40.15 µm^2^), representing a 116% increase compared to the low‐starch diet (9%) with 0.5 mm ingredient grinding size. Both starch levels (*F* (3, 98) = 17.92, *p*  < 0.001) and grinding size (*F* (1, 98) = 13.35, *p*  < 0.001) had a significant effect on the hepatocyte area (Figure 4).

**Figure 5:**
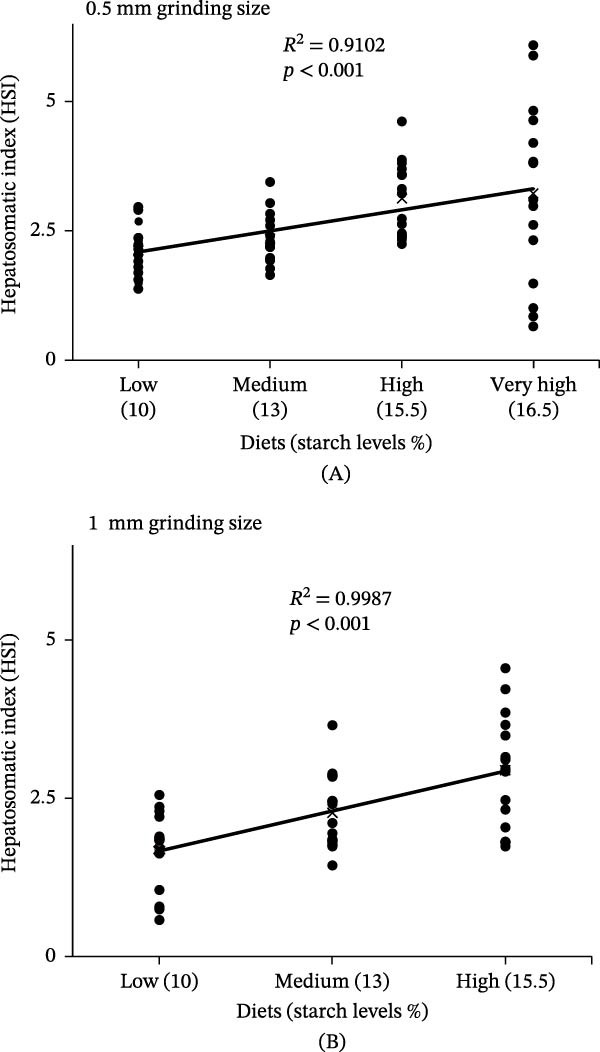
Correlation between hepatosomatic index and dietary starch levels in juvenile giant grouper fed diets produced using (A) 1 mm and (B) 0.5 mm ingredient grinding sizes.

Additionally, both dietary starch and grinding size affected the HSI of GG; however, neither dietary starch nor grinding size had an impact on the VSI (Table 2). The HSI of fish fed the very high‐starch diet (15.9%) increased by more than 50% compared to those fed the low‐starch diet (9%). Significant differences in HSI were found across starch levels (*F* (*3*, *98*) *= 10.37*, *p*  < 0.001), and between grinding sizes (*F* (*1*, *98*) *= 5.25*, *p*  < 0.05) (Table 2). Furthermore, a significant positive correlation (*R* ^2^ = 0.42, *p* < 0.001) was found between HSI and dietary starch levels (Figure 5).

Pearson’s correlation analysis revealed a significant positive relationship between HSI and hepatocyte area (*r* = 0.375, *p* < 0.001; Supporting Information 1: Figure S1). Similarly, villi length was positively correlated with lamina propria thickness (*r* = 0.237, *p* = 0.015; Supporting Information 2: Figure S2). Although the correlations were statistically significant, the relatively low *r*‐values indicate modest associations between these variables.

**Figure 6:**
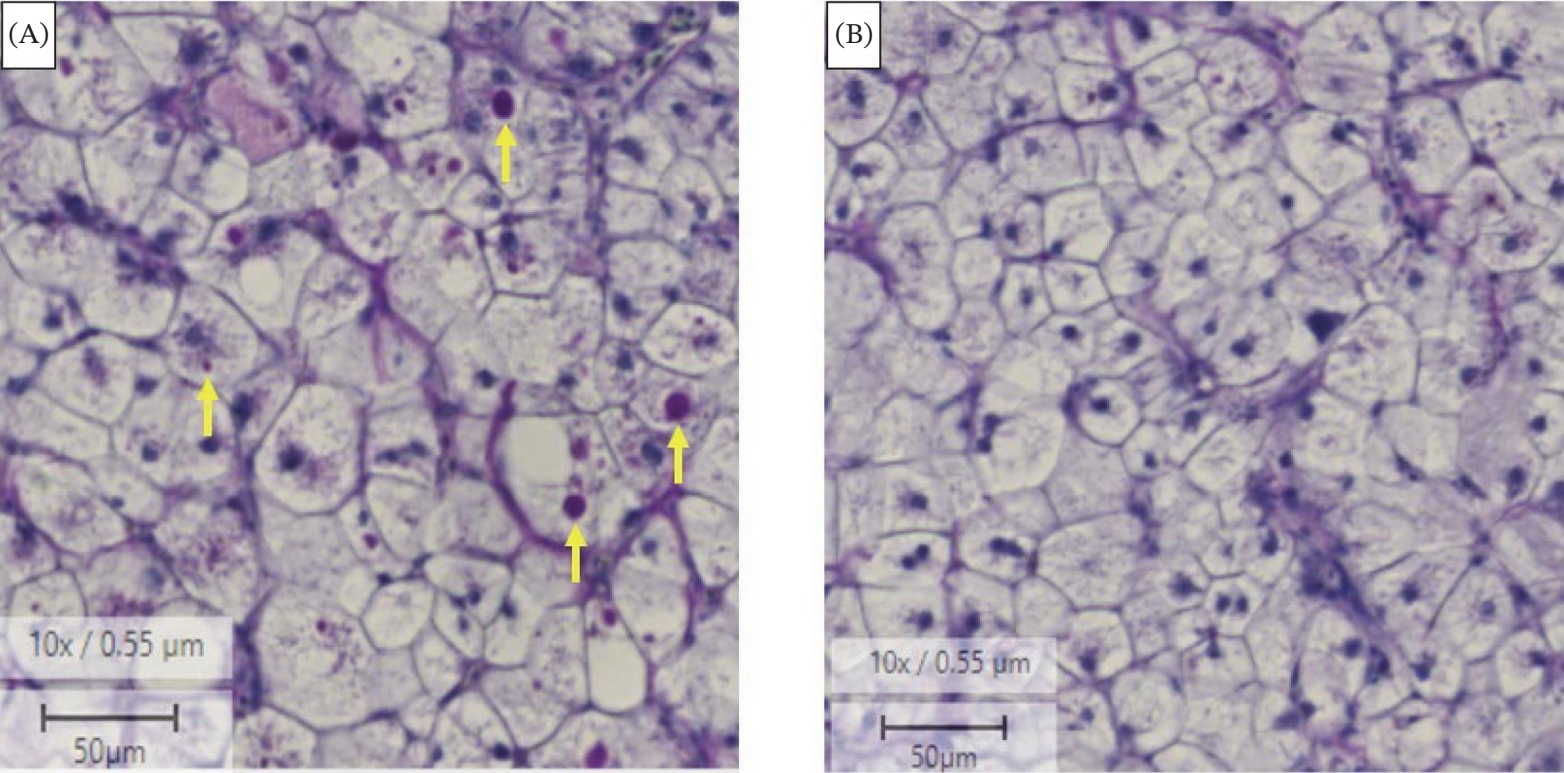
Photomicrograph of the hepatocyte of giant grouper *E. lanceolatus*. (A) Undigested glycogen accumulated in the liver of fish fed high‐starch diets. (B) Digested glycogen after treatment with diastase solution containing α‐amylase enzyme. Arrows indicate – glycogen.

The histological examination of GG hepatocytes revealed notable differences in glycogen content depending on the treatment. In fish fed 15% starch diets, photomicrographs demonstrated an accumulation of undigested glycogen within the liver cells, consistent with the elevated relative hepatic glycogen levels (GL; a.u.) quantified in Table 3 (Figure 6). Glycogen was confirmed as the cause of this change through its disappearance in a sequential section after treatment with a diastase solution containing the enzyme α‐amylase.

## 4. Discussion

In the present study, an ingredient grinding size of 0.5 mm negatively affected both the growth performance and FI in juvenile GG, contradicting the widely accepted benefits of finer raw material particle sizes in diets. While smaller grinding sizes are typically associated with improved nutrient digestibility and energy availability [40–42], our results indicate that the growth depression in the 0.5 mm group was primarily driven by reduced FI rather than enhanced nutrient utilisation.

Three possible explanations for the reduced FI in fish fed the 0.5 mm grinding‐size diet include improved nutrient digestibility, allowing sufficient energy intake without increased feed consumption [40–42], slower digesta transit rates, extending nutrient absorption [40–43] and potential negative factors such as an inflamed posterior intestine. However, these factors alone may not fully account for the observed slower growth, suggesting that a multifactorial interaction, possibly involving digestibility, metabolic costs and inflammation, may be responsible.

In addition to these physiological mechanisms, differences in pellet physical characteristics may also have contributed to the reduced FI observed in the 0.5 mm group. Finer grinding size can influence particle packing and starch–protein interactions during extrusion, potentially altering pellet hardness, durability, density and water stability, all of which affect feed physical quality [17, 44]. In carnivorous species such as giant grouper, feed texture and mechanical resistance can influence voluntary FI and feeding behaviour. It is therefore possible that structural differences associated with the finer grinding size affected feed acceptance. However, pellet physical properties were not directly measured in the present study, and this explanation should be interpreted cautiously. Future studies incorporating physical quality assessments of pellets would help clarify the contribution of these factors.

These findings indicate that while grinding size exerted a stronger influence on growth‐related parameters than dietary starch level, the combination of both factors modulated feeding behaviour, particularly at extreme starch inclusions.

In terms of intestinal morphology, increased dietary starch levels significantly widened the lamina propria thickness, consistent with findings in other species [45, 46]. This morphological response, together with the observed infiltration of granular cells, indicates an early inflammatory reaction. While such responses may initially support tissue defence, persistent lamina propria widening could compromise intestinal absorptive efficiency in longer‐term feeding trials. Thus, intestinal morphology changes in GG suggest that excessive dietary starch may impair gut function over time, even if short‐term growth performance remains relatively unaffected.

One potential strategy is the inclusion of short‐chain fatty acids (SCFAs), such as butyric and propionic acid, as feed additives. These compounds are known to support intestinal health and digestion by promoting the growth of beneficial gut bacteria and reducing inflammation [47]. Additionally, the use of probiotics and prebiotics may further enhance intestinal absorption and improve overall growth and feed conversion [48].

The liver morphometric analysis revealed a significant increase in HSI with higher dietary starch levels, where fish fed a very high‐starch diet (15.9%) showed a twofold increase compared to those on a 9% starch diet. This increase is strongly associated with glycogen deposition and hepatocyte area enlargement. Such enlargement is consistent with reports in rainbow trout [25, 49, 50] and hybrid grouper [18, 51, 52], supporting the view that carnivorous fish species have limited capacity to regulate high dietary carbohydrate intake.

Furthermore, no significant effects of dietary starch levels or grinding size were observed on the VSI of juvenile GG. This suggests that excess available energy from elevated dietary starch in groupers predominantly results in liver glycogen deposition, without any indication of energy being deposited as visceral fat.

Glycogen accumulation leading to liver enlargement has been documented in several studies [23, 49]. In aquaculture, such enlargement is often interpreted as a sign of metabolic stress [53, 54]. However, in GG, the enlargement observed may also reflect a physiological adjustment to increased carbohydrate availability, not necessarily a pathological state. In carnivorous fish species, limited capacity for carbohydrate utilisation can result in prolonged postprandial hyperglycaemia and enhanced hepatic glycogen storage when dietary starch levels are elevated [23, 27, 55]. Without measurements of plasma glucose concentration, hepatic glycolytic and gluconeogenic enzyme activities (e.g. glucokinase, phosphofructokinase and glucose‐6‐phosphatase), or other metabolic indicators, it is not possible to determine whether the observed glycogen deposition represents adaptive energy storage or metabolic dysregulation. Therefore, future studies incorporating blood biochemical parameters and hepatic enzyme activity assessments would provide valuable insight into the metabolic consequences of high dietary starch in giant grouper. Distinguishing between adaptive glycogen storage and maladaptive stress responses will require further biochemical and histopathological studies.

While dietary starch can provide some nutritional benefits, its use in aquafeed formulations for GG should be minimised to avoid potential adverse effects associated with intestinal inflammation and hepatic glycogen accumulation. Understanding the mechanisms underlying liver enlargement and intestinal inflammation will be essential in refining the nutritional requirements of GG. This knowledge will ultimately contribute to the development of cost‐effective, species‐specific aquafeeds that optimise both health and growth in this important aquaculture species.

## 5. Conclusions

In conclusion, this study demonstrates that ingredient grinding size and dietary starch levels significantly influence growth performance, FI and organ morphometrics in juvenile GG. A reduction in grinding size from 1 to 0.5 mm resulted in decreased SGR, while FI declined with increasing starch levels, particularly in finely ground diets. FCR, however, remained unaffected by either factor. Notably, higher dietary starch levels led to significant morphological changes in the liver and intestine, including enlarged hepatocytes and increased HSI. These findings highlight the importance of optimising dietary formulations, specifically grinding size and starch inclusion levels, to balance growth performance and physiological health in GG aquaculture. Future studies should focus on refining these dietary parameters to ensure the sustainable and effective culture of juvenile GG.

## Author Contributions

Albino M. Maveto conducted the experiment, collected the data, analysed the results and wrote the first draft. Leo Nankervis and Caroline Candebat supervised the student and reviewed the draft. Leo Nankervis acquired the funding and provided resources. Adrien F. Marc reviewed the draft. Leo Nankervis and Simon Kumar Das reviewed, edited and finalised the manuscript.

## Funding

This study was funded by the Australian Centre for International Agricultural Research (Grant FIS/2021/121). Open access publishing facilitated by James Cook University, as part of the Wiley ‐ James Cook University agreement via the Council of Australasian University Librarians.

## Ethics Statement

This study was conducted with approval from the James Cook University Animal Ethics Committee (A2713).

## Conflicts of Interest

The authors declare no conflicts of interest.

## Supporting Information

Additional supporting information can be found online in the Supporting Information section.

## Supporting information


**Supporting Information 1** Figure S1. Pearson correlation between hepatosomatic index (HSI) and hepatocyte area (µm^2^) in juvenile giant grouper (Epinephelus lanceolatus). The solid line represents the linear regression (*r* = 0.376, *p* < 0.001).

**Supporting Information 2** Figure S2. Pearson correlation between villi length (µm) and lamina propria thickness (µm) in juvenile giant grouper (Epinephelus lanceolatus). The solid line represents the linear regression (*r* = 0.237, *p* = 0.015).

## Data Availability

Data are available upon request from the authors.
