# Supplementary material for: Interactive Effects of Dietary Starch Level and Ingredient Grinding Size on Growth, Intestinal Health and Liver Condition of Juvenile Giant Grouper (Epinephelus lanceolatus)
Source: Aquac Nutr. 2026 May 9;2026:3101205. doi: 10.1155/anu/3101205 (PMC13157327; doi:10.1155/anu/3101205)
Supplement: Supplementary file 1 — Supporting Information 1 Figure S1. Pearson correlation between hepatosomatic index (HSI) and hepatocyte area (µm2) in juvenile giant grouper (Epinephelus lanceolatus). The solid line represents the linear regression (r = 0.376, p < 0.001). [file ANU-2026-3101205-s002.docx]

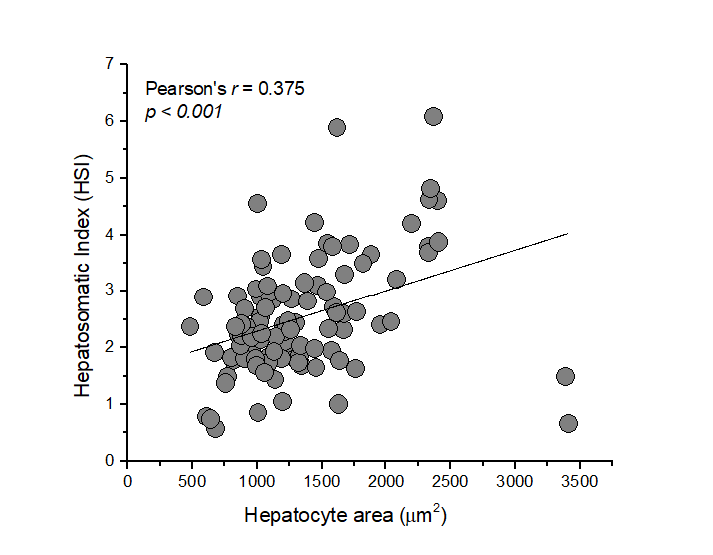


**Supplementary Figure S1**. Pearson correlation between hepatosomatic index (HSI) and hepatocyte area (µm²) in juvenile giant grouper (*Epinephelus lanceolatus*). The solid line represents the linear regression (r = 0.376, *p* < 0.001).
