# Supplementary material for: Interactive Effects of Dietary Starch Level and Ingredient Grinding Size on Growth, Intestinal Health and Liver Condition of Juvenile Giant Grouper (Epinephelus lanceolatus)
Source: Aquac Nutr. 2026 May 9;2026:3101205. doi: 10.1155/anu/3101205 (PMC13157327; doi:10.1155/anu/3101205)
Supplement: Supplementary file 2 — Supporting Information 2 Figure S2. Pearson correlation between villi length (µm) and lamina propria thickness (µm) in juvenile giant grouper (Epinephelus lanceolatus). The solid line represents the linear regression (r = 0.237, p = 0.015). [file ANU-2026-3101205-s001.docx]

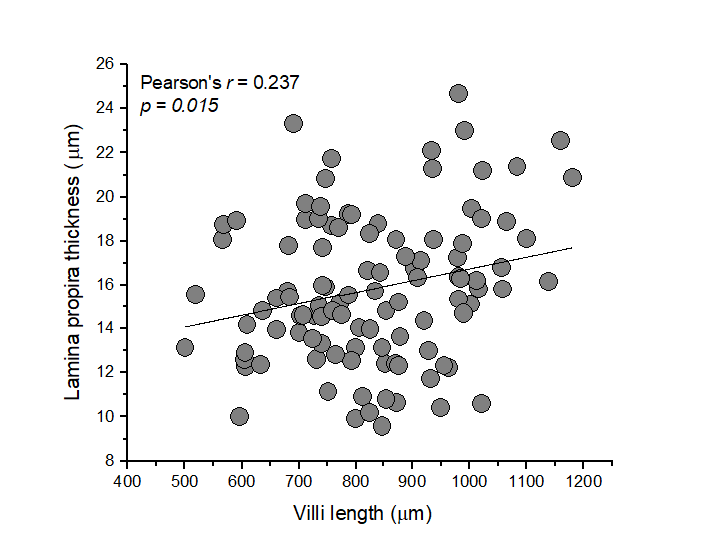


**Supplementary Figure S2.** Pearson correlation between villi length (µm) and lamina propria thickness (µm) in juvenile giant grouper (*Epinephelus lanceolatus*). The solid line represents the linear regression (r = 0.237, *p* = 0.015).
